# Establishing a quality management framework for commercial inoculants containing arbuscular mycorrhizal fungi

**DOI:** 10.1016/j.isci.2022.104636

**Published:** 2022-06-18

**Authors:** Matthias J. Salomon, Stephanie J. Watts-Williams, Michael J. McLaughlin, Heike Bücking, Brajesh K. Singh, Imke Hutter, Carolin Schneider, Francis M. Martin, Miroslav Vosatka, Liangdong Guo, Tatsuhiro Ezawa, Masanori Saito, Stéphane Declerck, Yong-Guan Zhu, Timothy Bowles, Lynette K. Abbott, F. Andrew Smith, Timothy R. Cavagnaro, Marcel G.A. van der Heijden

**Affiliations:** 1 The Waite Research Institute and The School of Agriculture, Food and Wine, The University of Adelaide, Waite Campus, PMB1 Glen Osmond, SA 5064, Australia; 2 University of Missouri, Division of Plant Sciences, Columbia, MO 65211, USA; 3 Global Centre for Land-Based Innovation, Hawkesbury Institute for the Environment, Western Sydney University, Penrith, SA 2747, Australia; 4 INOQ GmbH, Schnega 29465, Germany; 5 Université de Lorraine, INRAE, UMR Interactions Arbres/Microorganismes, Centre INRAE Grand Est-Nancy, Champenoux, France; 6 Beijing Advanced Innovation Center for Tree Breeding by Molecular Design, Beijing Forestry University, 100083 Beijing, China; 7 The Institute of Botany, Czech Academy of Sciences, Zamek 1, 25243 Pruhonice, Czech Republic; 8 State Key Laboratory of Mycology, Institute of Microbiology, Chinese Academy of Sciences, No. 3 1st Beichen West Rd., Chaoyang District, Beijing 100101, China; 9 Graduate School of Agriculture, Hokkaido University, Sapporo, Hokkaido 060-8589, Japan; 10 Iwate University, Morioka, Iwate 020-8550, Japan; 11 Laboratory of Mycology, Earth and Life Institute, Université catholique de Louvain, Croix du Sud 3, 1348 Louvain-la-Neuve, Belgium; 12 Key Laboratory of Urban Environment and Health, Institute of Urban Environment, Chinese Academy of Sciences, 1799 Jimei Road, Xiamen 361021, China; 13 University of Chinese Academy of Sciences, 19A Yuquan Road, Beijing 100049, China; 14 Department of Environmental Science, Policy and Management, University of California Berkeley, Berkeley, CA 94720, USA; 15 UWA School of Agriculture and Environment and UWA Institute of Agriculture, The University of Western Australia, Perth, WA, Australia; 16 Plant-Soil-Interaction Group, Institute for Sustainability Science, Agroscope, Zürich, 8046 Switzerland; 17 Department of Plant and Microbial Biology, University of Zurich, Zürich 8008, Switzerland

**Keywords:** Environmental science, Environmental health, Biological sciences, Biotechnology, Plant biology, Interaction of plants with organisms, Plant nutrition

## Abstract

Microbial inoculants containing arbuscular mycorrhizal (AM) fungi are potential tools in increasing the sustainability of our food production systems. Given the demand for sustainable agriculture, the production of such inoculants has potential economic value and has resulted in a variety of commercial inoculants currently being advertised. However, their use is limited by inconsistent product efficacy and lack of consumer confidence. Here, we propose a framework that can be used to assess the quality and reliability of AM inoculants. First, we set out a range of basic quality criteria which are required to achieve reliable inoculants. This is followed by a standardized bioassay which can be used to test inoculum viability and efficacy under controlled conditions. Implementation of these measurements would contribute to the adoption of AM inoculants by producers with the potential to increase sustainability in food production systems.

## Highlights

• Inoculants containing AMF are a potential tool for sustainable agriculture
• Their use is limited by inconsistent product efficacy
• We propose a quality framework to improve the quality of these inoculants
• This framework consists of required quality criteria and a standardized bioassay

## Graphical abstract

**Figure:**
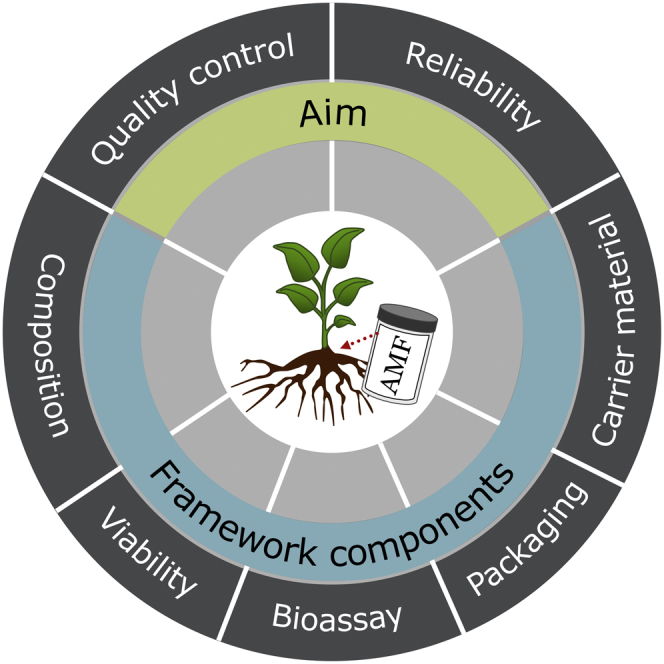

## Introduction

One of the major challenges of the 21^st^ century is the sustainable production of food for an ever-growing population, which is expected to reach 9.7 billion people by 2050 (United Nations, 2019). Increases in yields of food production systems over the last two centuries have been heavily reliant on chemical pesticides and mineral fertilizers (Liu et al., 2015). However, these products are part of the world’s most energy-intensive production processes and are often dependent on finite resources such as phosphorus (P) fertilizers (Woods et al., 2010). Many crops have a low P fertilizer use efficiency, resulting in low recovery of applied fertilizer in plants (Baligar et al., 2001). The extensive use of fertilizers in food production systems is a major factor contributing to agricultural global greenhouse gas emissions (Vermeulen et al., 2012), and can have severe adverse effects on biodiversity and environmental sustainability (Steffen et al., 2015). Furthermore, there is evidence that agrochemical-based food production systems have reached a plateau in productivity (Lobell et al., 2011). Projections show that current yield trends will not meet the food demand for future decades without changes in diet or reductions of food waste (Cassidy et al., 2013; Ray et al., 2013). Other pressing issues include the development of pesticide resistance (Gould et al., 2018), the emergence of new crop pathogens (Fones et al., 2020), and increasing consumer demand for pesticide-free food (Rana and Paul, 2017). There is rapidly emerging interest to reduce our agricultural footprint and reliance on agrochemicals through the use of biostimulants, including microbial inoculants (Abbott et al., 2018). Commercial microbial inoculants include the highly successful rhizobia products (Howieson and Dilworth, 2016) and other selected generalist organisms, such as *Bacillus sp*. or *Trichoderma sp*., that seek to improve plant vigor and have significant potential to reduce the demand of agrochemicals (Berruti et al., 2016; Owen et al., 2015). These microbial products have the potential to increase farm productivity and yield resilience for sustainable food production (Singh et al., 2020); their use underpins various global challenges and sustainable development goals, such as food safety, food security, and climate change mitigation (D’Hondt et al., 2021).

One group of well-studied symbionts is arbuscular mycorrhizal fungi (AMF) which colonize roots and provide nutrients in exchange for photosynthates. AMF have been shown to improve the uptake of essential plant nutrients, such as P, zinc, and nitrogen (Smith and Read, 2008; van der Heijden et al., 2015). At the same time, they may increase plant resistance toward pathogens (Jung et al., 2012) and other abiotic stresses, such as drought or salinity (Plouznikoff et al., 2016) (see Table 1). AMF follow a cosmopolitan distribution and can be found in almost all ecosystems (Öpik et al., 2006). However, their natural abundance can be diminished by common agricultural practices, including the application of fertilizers (Cheng et al., 2013), soil disturbance (van der Heyde et al., 2017), or selection of cultivars that associate less with AMF (Zhang et al., 2019). Conversely, AMF populations can also be bolstered using management practices such as cover crops (Bowles et al., 2017) and principles of organic farming (Verbruggen et al., 2010). Where these practices are not applicable, the *in situ* use of AMF inoculum has been shown to increase arbuscular mycorrhizal root colonization and yield resilience (Giovannini et al., 2020; Hijri, 2016).

**Table 1:** Overview of potential mycorrhizal benefits toward plant growth and ecosystems

Benefits	Reference
**Plant**	

Improved uptake of minerals, especially phosphorus, copper, and zinc.	Watts-Williams et al., 2013
Increased plant biomass and yields.	Rocha et al., 2019a, 2019b; Zhang et al., 2019
Improved water uptake, osmotic regulation, and drought resistance.	Augé, 2001
Improved resistance against soil salinity.	Evelin et al., 2019; Fileccia et al., 2017
Increased plant metabolite production.	Zeng et al., 2013
Protective effects toward soil contamination and adverse soil physiochemical characteristics.	Gamalero et al., 2009; Lenoir et al., 2016
Induction of systemic pathogen resistance.	Pieterse et al., 2014
Protective effects against nematodes and root diseases.	Harrier and Watson, 2004
Increased nitrogen fixation in legumes.	Kafle et al., 2019; Püschel et al., 2017

**Ecosystem services**	

Soil aggregation, improved soil structure, and carbon sequestration.	Rillig and Mummey, 2006; Wilson et al., 2009
Reduced nutrient leaching.	Cavagnaro et al., 2015
Interaction and driving force of microbial activities.	Barea et al., 2002
Reduced greenhouse gas (N_2_O) emissions from soils.	Bender et al., 2014
Common mycorrhizal network between plants for allocation of nutrients, seedling establishment, and plant-to-plant interactions.	Van der Heijden and Horton, 2009

## Status quo

With the global economic value for microbial inoculants expected to reach $11.45 billion USD by 2026 (Stratistics Market Research Consulting, 2018), an increasing number of commercial AMF inoculants have been released onto the market in the last few decades (Benami et al., 2020; Vosátka et al., 2008). Retail markets in most countries offer a variety of commercial AMF inoculants which are available for amateur and professional applications alike (Bitterlich et al., 2020; von Alten et al., 2002). One meta-analysis of 28 AMF manufacturers showed that over 90% of the 68 AMF products are currently provided in a solid-state and 10% as liquid formulation. All analyzed products used species within the Glomeraceae, of which *Rhizophagus irregularis* (39%), *Funneliformis mosseae* (21%), and *Claroideoglomus etunicatum* (16%) are most frequently used. Two third of the products used a conglomerate of AMF species rather than a single species. About 20% of the products include other beneficial microorganisms (Basiru et al., 2020).

However, for many years, the global market for agricultural microbial inoculants has been lagging behind the expectations that followed from scientific findings in laboratory or controlled environments. One of the reasons for this is the inconsistent results of microbial inoculants, including AMF, when applied under various field conditions (Bender et al., 2019; Singh and Trivedi, 2017). For AMF, this could be caused by environmental factors, such as incompatible symbionts that are not adapted to soil and climate conditions, but also technical reasons, such as poor product quality. For most consumers, it is impossible to verify the quality of AMF inoculants due to the need for laboratory facilities and expertise. In addition, many commercial inoculants incorporate a variety of (non-AMF) plant-growth-promoting microorganisms, biological additives, and/or plant nutrients. Often, these additives are not clearly disclosed, and positive plant growth effects may be falsely attributed to AM colonization (Salomon et al., 2022). In addition, the commonly used *in vivo* production method for AMF inoculum can introduce unwanted contaminants such as nematodes, weeds, algae, or saprophytes when quality control systems are not in place (Hart et al., 2017; von Alten et al., 2002). Another concern relates to the supply chain, which is prone to unfavorable or prolonged storage conditions, impacting the viability of inoculants.

Mandatory quality control of commercial AMF inoculants is sparse or non-existent in most countries, which makes it voluntary for producers to undertake such measurements. Previous studies from multiple countries showed consistently that ineffective AMF inoculants are common rather than an exception (Faye et al., 2013; Tarbell and Koske, 2007). In a recent study by Salomon et al. (2022), 25 AMF products from Australia and Europe were tested under greenhouse conditions. Over 80% of the commercial AMF inoculants failed to induce arbuscular mycorrhizal root colonization in sterilized soils under AMF-favorable conditions.

Quality control mechanisms that seek to regulate AMF inoculants were established in Japan by the *Soil Productivity Improvement Act* in 1996 (Saito and Marumoto, 2002). This legislation was implemented as a reaction toward Japan’s first wave of agricultural microbiology in the 1990s, during which several agrochemical companies released AMF inoculants. The Japanese Government approved AMF inoculants alongside official criteria for overseeing the quality of such products. A standard bioassay protocol was introduced which governed mandatory testing and labeling guidelines (see supplemental information). Ongoing research confirmed the reliability of domestic AMF producers (e.g. Niwa et al. (2018)), indicating that the introduced measurements were efficient.

A more recent legislative quality management of AMF products is the amendment of the EU fertilizer regulation 2019/1009, which took effect in April 2019. To date, the European standardization committee CEN TC 455 ″plant biostimulants" is establishing standard methods for the product certification of AMF inoculants. These standards will be tested and verified in Europe-wide ring tests, performed by independent laboratories. The focus of these methods is on the quantification of viable microorganisms in the products, and the validation of claimed benefits through standardized tests (e.g. increased nutrient uptake, abiotic stress resistance, and plant growth promotion).

Various methods are available to assess AMF spore viability. Common methods involve spore viability staining (Meier and Charvat, 1993), *in vitro* germination (Maia and Yano-Melo, 2001), or the most probable number (MPN) method (Porter, 1979). Spore viability staining is a relatively fast approach, in which AMF spores are extracted and treated with dehydrogenase-activated stain. Metabolic active spores show a color response and can be separated from inactive spores, which are considered non-viable. However, this method has been shown to produce inconsistent results and spore viability might differ from actual root colonization under realistic conditions (Meier and Charvat, 1993). *In vitro* spore germination tests are another relatively fast approach to assess spore viability. Extracted spores are surface-sterilized and placed in Petri dishes for visual confirmation of successful germination. Like the spore viability staining, results of *in vitro* germination tests might not correlate with root colonization under *in situ* conditions. Different germination rates are found with different *in vitro* media (Maia and Yano-Melo, 2001). Furthermore, this method requires knowledge about the use of aseptic techniques, and the surface sterilization of AMF spores is a delicate balance between de-activating contaminants and preserving spore viability (Declerck et al., 2005). The MPN method involves spore counting of the original AMF inoculum, which is then diluted into aliquots. Plants are grown in all aliquots and the MPN is determined based on the original spore count and the presence of root colonization in the aliquots (Porter, 1979). This method is labor intensive as all aliquots require repetitions to reduce variability. Furthermore, all of the presented methods require spore extraction from the inoculum. This can be problematic in carrier materials with porous spaces, such as expanded clay or perlite, and can underestimate the actual number of AMF spores (Louis Mercy, INOQ, personal communication). Also, the results may be inaccurate if the inoculum consists of high numbers of root fragments or hyphae, which are not considered by these methods.

## New proposed framework

**Figure 1:**
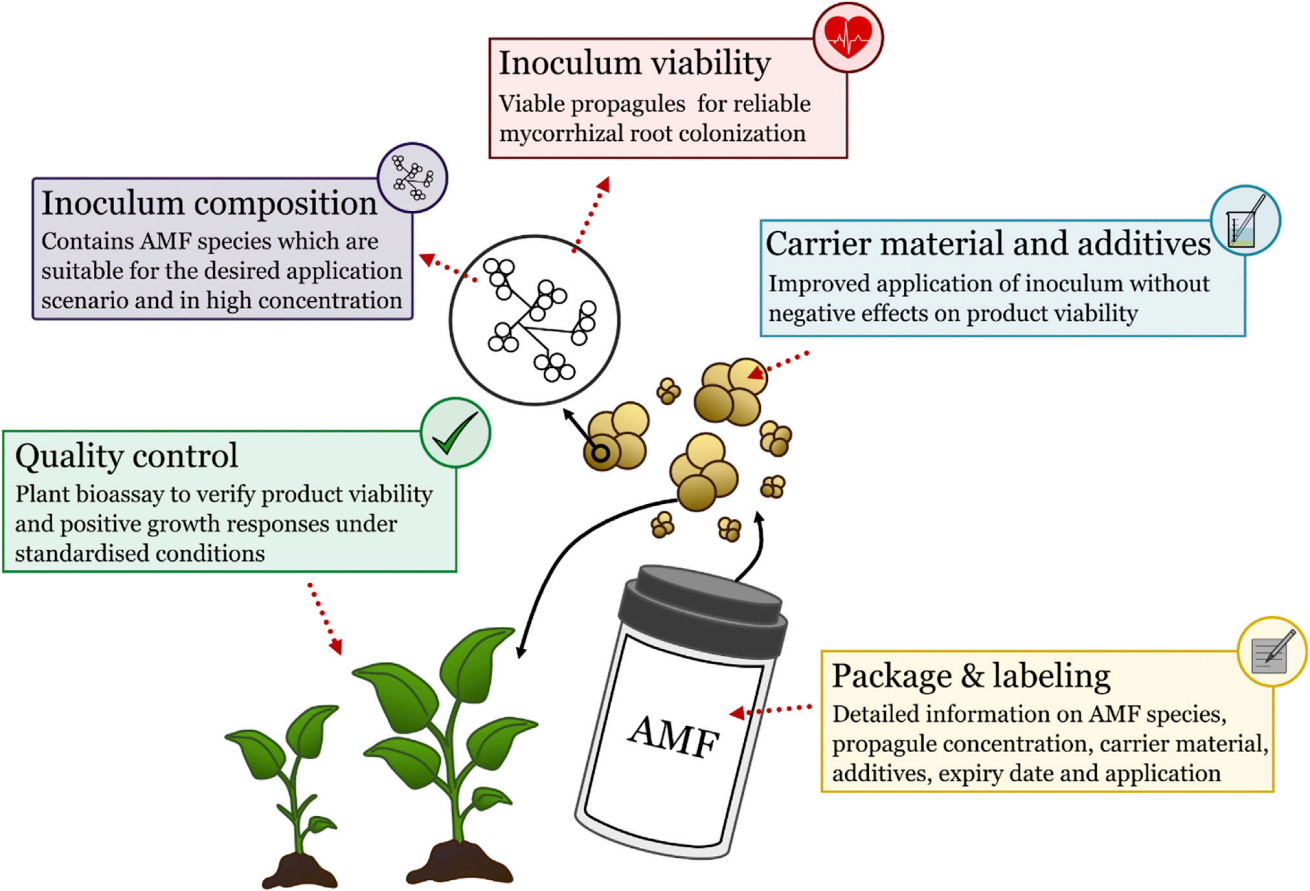
Quality criteria for microbial inoculants containing arbuscular mycorrhizal fungi.

Building on the efforts in Japan and the EU, the authors propose the development of a general quality management framework for commercial AMF inoculants. This framework takes into account both economic requirements and validity of results under applied scenarios. We identified essential quality criteria that need to be met by producers to ensure working AMF inoculants (see Figure 1 and Table 2). As a first step, we focus solely on the most basic quality criteria for AMF inoculants which can be summarized as:

• Occurrence of viable propagules (spores, hyphae, and AMF-colonized root pieces) that result in arbuscular mycorrhizal root colonization under controlled conditions
• Absence of plant pathogens and other contamination
• Product formulation for facilitated inoculum application (e.g. pure AMF blends, carrier materials, or solutions)
• Detailed description of AMF species, additives, storage criteria, and application procedures.

**Table 2:** Proposed quality criteria and quality control for AMF inoculants that need to be met by producers

**Quality criteria**	

Inoculum composition and viability	• Inclusion of a generalist AMF species • Exemption applies for specialized inoculum for specific host plants • Free of plant pathogens, weeds, and other contaminants • Fast distribution channels to end-consumer, e.g., via selected retailers or direct selling.
Carrier material	• Facilitates application of inoculum • Only suitable additives that do not interfere with the mycorrhizal development
Package label	• Propagule composition (AMF isolates) • Carrier material and other additives • Plant-available nutrients (NPK) • Batch number • Production and expiration date • Instructions on storage and application • Documented evidence of root colonization (including picture) and plant growth stimulation on the producer’s website

**Quality control**	

	• Confirmed root colonization in standardized bioassay • Confirmed plant growth stimulation in standardized bioassay • Visual confirmation of the absence of unwanted contaminants, such as weeds or plant pathogens

**Table 3:** Specification for the standardized in vivo bioassay

Host plants	Maize (*Zea mays*) or Sorghum (*Sorghum bicolor*)	Leek (*Allium porrum*)
Growth period (Starting from seedling emergence or transplanting of seedlings)	6 weeks	10 weeks
Minimum pot size	2 liters	1 liter
Plants per pot	1	1
Minimum replicates per treatment	6	
Soil: sand/vermiculite dilution (using fine sand or vermiculite and agricultural soil that is typical for the region where the inoculant is tested)	1:9	
Substrate sterilization	Autoclavation for 60 min at 121°C or steaming for 60 min at 80°C or gamma sterilization	
Phosphorus addition	20 mg P kg^−1^ substrate, in form of 88.4 mg CaH_2_PO_4_ kg^−1^ substrate	
Nutrient solution (Long Ashton -P) *(lacking phosphorus)*	Weekly, 20 mL per L^−1^ substrate	Every second week, 20 mL per L^−1^ substrate
Watering *Reverse Osmosis or distilled H*_*2*_*O*	Every second day to field capacity	
Temperature	18°C (night) to 30°C (day)	
Daylight average light intensity	>600 μmol m^−2^ s^−1^	

These criteria are to be validated using a standardized *in vivo* bioassay which provides data about mycorrhizal effects on plant biomass and colonized root length (see supplement S2). This plant growth bioassay is a low-cost method for validating propagule viability in a plant substrate. It provides additional information regarding the mycorrhizal growth effect (MGR) under controlled conditions and potential contamination with plant pathogens, be it through visual symptoms, reduced plant growth, or plant mortality.

The proposed framework could be adapted by regulatory agencies for product evaluation. Certification labels could be introduced for compliance by commercial AMF manufacturers. Such control measurements will lead to increased consumer confidence, thereby supporting the adoption of AMF inoculants by primary producers.

## Basic quality criteria

### Inoculum composition and viability

The selection of AMF species should be appropriate for the desired application scenario. If the AMF inoculant is intended for general use, it should contain at least one generalist species that is able to colonize a broad range of host plant species. Generalist AMF species that are widely used for commercial and scientific purposes are *Rhizophagus sp*. and *Glomus sp*. (Öpik et al., 2006). More selective application scenarios often require specialized AMF species, such as the use of *Acaulospora sp*. for acidic soils (Aguilera et al., 2015). Transparency is required about the source location, identification, and selection of the used AMF strain and should be documented accordingly, for example on the product or the producer’s website. Ideally, the selected AMF isolates are deposited in recognized collections under “safe deposit”, meaning that they cannot be released without the producer’s consent. This would ascertain correct identification of the isolates and their safe keeping in specialized facilities for future purposes.

The inoculant should contain enough viable propagules to achieve AM root colonization. High concentrations of viable propagules are particularly important to account for the declining germination rate of AMF propagules after longer product storage periods (Ruiz-Lozano and Azcon, 1996). Consequently, dosage recommendations should account for decreased propagule viability over time and contain defined margins. Inoculum viability is highly variable between AMF isolates (Smith and Read, 2008) and the host plant used for inoculum production (Dietrich et al., 2020). Producers need to verify the expiration date for their specific AMF isolates and production method. This can be done using the proposed standardized bioassay as outlined in Section 5.

AMF inoculants should be free of plant pathogens and other harmful contaminations. To this date, most inoculants are produced *in vivo* on host plants such as sorghum or maize (Berruti et al., 2016). In this case, host plants are grown in sterilized substrates and inoculated with the desired AMF species. These production systems naturally include a range of microorganisms associated with the AMF propagules. However, none of those microorganisms should be pathogenic to the host plant or its environment. A variety of molecular tests are available to confirm the absence of plant pathogens (Ophel-Keller et al., 2008). Such testing would not be required for *in vitro* produced propagules, where AMF are propagated under monoxenic conditions in a laboratory environment. Various initiatives have been reported which may facilitate the large-scale production of monoxenic AMF inoculants in the nearer future (Gargouri et al., 2021; Ijdo et al., 2011; Sugiura et al., 2020). To date, only a few AMF species can be produced *in vitro* and on a large scale, making *in vivo* methods the preferred choice for many companies. This, however, might change in the near future due to technical advancements.

Selected AMF isolates are used in a standardized bioassay to evaluate their MGR. The bioassay uses model mycorrhiza-responsive crops such as sorghum *(Sorghum bicolor)*, maize *(Zea mays)*, or leek *(Allium porrum)* which are grown under standardized conditions (see section 5) (Tran et al., 2019). The plant substrate for this bioassay is predominantly made from inert materials (sand or vermiculite) to provide a certain level of homogeneity. It contains relatively low concentrations of P, to facilitate mycorrhizal root colonization. This bioassay also serves to uncover the potential presence of plant pathogens which would negatively affect the MGR. However, the main purpose of this bioassay is the validation of propagule viability and successful root colonization. The resulting MGR and the used host plant is documented and reported on the package label (see 4.3). This bioassay should be performed on a subset of the finished inoculum and then annually thereafter.

### Carrier materials

Dispersal of AMF propagules in a carrier material should facilitate the application of the inoculant without negatively affecting its viability. Various carrier material technologies are available for agricultural applications or environmental restoration, such as algal or polymeric beads (Vassilev et al., 2005), liquid solutions (Malusá and Vassilev, 2014), biochars (Sashidhar et al., 2020), or seed coatings (Rocha et al., 2019a; 2019b). Propagules can be dispersed in coarse material, such as calcinated clay to facilitate handling (Vassilev et al., 2005). The material should be homogenous so that AMF propagules can be dispersed evenly.

If biological or chemical additives are incorporated into the inocula, they need to work synergistically, or at least not reduce AMF colonization. Compounds that have been successfully tested in combination with AMF include various plant-growth-promoting microorganisms (Wu et al., 2005) and biological compounds, such as chitin or humic acids (Gryndler et al., 2003). Additives such as mineral fertilizers should not suppress the AM root colonization and need to be labeled appropriately.

### Package and labeling

AMF inoculants should be stored in a water- and light-proof container for improved propagule viability. Care must be taken during packaging to ensure that the propagules are undamaged and inoculum viability is maintained. Distribution channels between producers and consumers must avoid unfavorable conditions which could damage the propagules, such as prolonged storage times or extreme temperatures below 4°C and above 28°C (de Santana et al., 2014). Certain AMF strains can also germinate at cooler temperatures, which is an important consideration when developing a commercial inoculant (Carvalho et al., 2015). The viability and germination response of the selected strain needs to meet the available distribution channels and contain high viability after arriving at the consumer.

Where the product label does not provide sufficient space, information can be provided *via* additional product sheets or online. The package labeling must include all necessary information about the inoculum content (propagule composition and concentration), production method (*in vivo* or *in vitro*), additives, plant-available nutrients, batch number, production and expiration date, instructions on storage and application, and information about quality measures.

AMF inoculant producers should provide the data from the latest standardized *in vivo* bioassays (see section 5 and supplement S2) and any further conducted quality control measurements. This report should contain: 1) visual proof of root colonization by AMF under defined conditions, 2) the calculated MGR after inoculation compared to the non-inoculated control, 3) information about the used host plant, and 4) disclaimer text that results are context-dependent and may vary. Such information is provided on the company’s website, and regularly updated. It should also provide a transparent documentation about the used AMF strains and their original location, as well as identification and selection processes.

## Quality control: bioassay

Mycorrhizal inoculants should be tested in a standardized bioassay under controlled conditions (see supplement S2). Rather than focusing on the broader ecological and plant physiological advantages of AMF, the proposed bioassay is designed to control the minimum requirements for commercial AMF inoculants. The aim of this bioassay is to assess whether inoculants contain viable propagules and colonize selected host plants in sterilized substrates under controlled conditions. This bioassay provides additional information regarding potential contamination with plant pathogens, which are reflected in the MGR or which can be visually identified. The inoculants are tested under AMF-favorable conditions that include mycorrhizal-responsive host plants (maize, sorghum, or leek) which are grown under low concentrations of plant-available P (see Table 3). The desired outcome at the end of the bioassay is a positive growth response and a significant AM root colonization of at least 20% colonized root length, according to the proposed methodology in supplement S2. This measurement is only used as an indicator for inoculum viability and should not be advertised otherwise.

## Conclusion and future perspective

Microbial inoculants are an essential building block for resilient and sustainable food production systems. However, the current market requires intervention to break the cycle of unreliable products and skeptical consumers. The framework proposed here is intended as a starting point; it addresses necessary quality criteria and quality control measurements that can be used to improve the adoption of AMF inoculants. At present, the framework focuses on minimum requirements with the potential for modifications or intensification in the future. This intensification could be guided by scientific research focusing on the application of AMF inoculants under commercial conditions and any ecological consequences. More research and actions are required to address the following issues:

• Developing AMF application models to predict inoculation success and yield responses relative to environmental and farming conditions. This allows farmers to decide if the application of commercial AMF inoculants is economic and ecological viable.
• Understanding the establishment of introduced AMF under field conditions and its effects on indigenous AMF communities (Hart et al., 2017). This includes the molecular validation that introduced AMF are colonizing host plant roots or enhance AMF root colonization.
• Continuous efforts in evaluating the potential hazards of widespread AMF inoculant use.
• Providing services to analyze roots for arbuscular mycorrhizal colonization after the application of commercial inoculants to verify its establishment.
• Development of advanced production methods to achieve highly concentrated and contaminant-free inoculants (Gargouri et al., 2021; Tanaka et al., 2022).
• Evaluation of any new production methods regarding their effects on AMF functioning and genetic stability (Kokkoris and Hart, 2019).

To balance economic requirements, the proposed framework could be modified to be performed only every few years, with faster methods of quality control in between (e.g., spore staining). This, and further modifications, are subject to the cooperation between regulatory agencies and producers.

The framework proposed here is a first step toward the regulatory-backed improvement of AMF inoculants by ensuring basic quality criteria. It could be adapted via various pathways, such as an open partnership between companies, regulatory agencies, and primary producers. Major AMF producers need to be included during the implementation process to ensure its practicality and widespread adoption. Important discussion points for the legal adaptation include more specific mechanisms of certification, such as method standardization. Another important topic is the role of testing organization and the cost distribution between producers and regulatory agencies. Companies which adhere to the proposed requirements and provide transparent information about their production process would gain credibility with potentials for certification by an appropriate regulatory body. In return, primary producers could provide feedback for review by the companies during future product development. If the proposed minimum requirements for commercial AMF inoculants are met in a systematic way, we believe the growth of this industry will be significantly increased with the potential to increase sustainability in food production systems. We also highlighted that ongoing research and development is required to further improve the quality and efficiency of commercial AMF inoculants. It is important that safety assessments precede the inoculum production to avoid ecological damage and to guarantee that the widespread use of AMF inoculants yields in improved ecosystem functioning and plant growth.

## Supplemental information

Document S1. Texts S1–S3
