# Supplementary material for: Establishing a quality management framework for commercial inoculants containing arbuscular mycorrhizal fungi
Source: iScience. 2022 Jun 18;25(7):104636. doi: 10.1016/j.isci.2022.104636 (PMC9254352; doi:10.1016/j.isci.2022.104636)
Supplement: Document S1. Texts S1–S3 [file mmc1.pdf]

iScience, Volume *25*

Supplemental information

Establishing a quality management framework

for commercial inoculants containing

arbuscular mycorrhizal fungi

Matthias J. Salomon, Stephanie J. Watts-Williams, Michael J. McLaughlin, Heike Bücking, Brajesh K. Singh, Imke Hutter, Carolin Schneider, Francis M. Martin, Miroslav Vosatka, Liangdong Guo, Tatsuhiro Ezawa, Masanori Saito, Stéphane Declerck, Yong-Guan Zhu, Timothy Bowles, Lynette K. Abbott, F. Andrew Smith, Timothy R. Cavagnaro, and Marcel G.A. van der Heijden

Supplementary information

Supplementary text S1

Standard bioassay protocol for AMF inoculants in Japan

Excerpt from Soil Productivity Improvement Act (Law No.34 of 1979, amended in 1996)

Ministry of Agriculture, Forestry and Fisheries, Japan

1) Preparation of growth medium: Apply a standard amount of product (inoculum) to 50 cm^3 vermiculite and sow seeds of an assay plant.
2) Growth conditions: Grow the plants at 25˚C under a lighting condition of 15,000 – 20,000 lx (16 h light / dark cycle) for 4 weeks.
3) Assessment of mycorrhizal colonization:
   The roots are detached from the shoots, washed and cleared in 10% (w/v) KOH at 90˚C. Roots are then soaked in 5% (w/v) HCl for 10 min at room temperature and stained with 0.1% (w/v) aniline blue or trypan blue at 90˚C for 30 min.

   The stained roots are spread to a Petri dish with 1 cm grid lines, and the presence and absence of colonization are counted using the intersect gridline method (McGonigle et al., 1990). More than 100 intersections per sample are to be counted in three replication samples.

   Percentage of colonization is calculated as follows:

$$Colonization\ [\%] = \frac{No.\ colonized\ intersections}{No.\ total\ intersections} \times 100$$

4) Quality criteria for AMF inoculants: Root colonization ≥ 5%
5) Mandatory information on the product label
   - Colonization [%] in bioassay and used host plant
   - Used carrier material
   - Applicable and non-applicable plants
   - Expiration date

Supplementary text S2

Standard *in vivo* bioassay

Here, we describe a standardized *in vivo* bioassay for the evaluation of AMF inoculum viability and its effect on plant growth. Further specifications for this protocol are given in Table 3. Detailed instructions are available at: https://dx.doi.org/10.17605/OSF.IO/R9WGN.

1) The soil for this bioassay contains low concentrations of plant-available P which are sufficient to allow healthy plant growth, without suppressing mycorrhizal root colonization. One practical solution is the reduction of soil P through mixing soil with an inert substrate like sand or vermiculite and the re-introduction of P in the form of slow-releasing monocalcium phosphate (CaH_2PO_4). Other essential plant nutrients, such as nitrogen, potassium and micronutrients, are added throughout the bioassay in the form of nutrient solutions that are lacking P (modified Long Ashton -P, see Suppl. S3). The soil is sterilized to inactivate any native AMF propagules.
2) The inoculum is tested against a non-inoculated control group to quantify the MGR. Each treatment has a minimum of 6 biological replicates to allow the statistical testing of effects. For the inoculated group, the inoculum is applied as recommended by the manufacturer. Suitable host plants are added, either as seeds or seedlings. The soil is regularly watered, to near field capacity and the nutrient solution is applied weekly or biweekly. All groups are treated identically in terms of dry soil weights, water and fertilizer applications, and homogenous seedling materials.
3) At the end of the bioassay, plants are destructively harvested by carefully removing the plants from the pots and washing the soil off the roots. A subsample of about 300 mg fresh roots is taken and stored in 50% EtOH. The shoots and roots are separated, dried at 65 °C for at least 48 hours and the dry weights recorded. The MGR can be calculated as followed:

$$\frac{[\text{Biomass (inoculated)} - \text{Biomass (control)}]}{\text{Biomass (control)}}$$

The subsampled roots are stained following the ink-vinegar method as described by Vierheilig et al. (1998) and visualized in the book by Brundrett et al. (1996) First, the roots are washed with water and cleared in 10% KOH, either at room temperature for 3-4 days or for 10-15 minutes at 80 °C. The exact time depends on the plant species, root thickness and root pigmentation. Roots are fully cleared when only the cell wall and cell membrane remain visible under a dissecting microscope. Roots are washed again with water and stained in a 10% ink and 90% vinegar solution for 15 minutes at 65 °C. After staining, roots are washed under water and de-stained for one day in an acidified water solution, containing 2% household vinegar (approx. 5% acetic acid). Roots are now ready for examination or can be stored in a 50% glycerol solution. The colonized root length can be determined following the grid-line intersect technique described by (McGonigle et al., 1990).

Supplementary text S3

Formulation for modified Long Ashton nutrient solution lacking P (Cavagnaro et al., 2001)**. Concentrations are referring to the chemical compound rather than the element.**

Macronutrients		Micronutrients	
Potassium sulphate (K_2SO_4)	2 mM	Boric acid (H_3BO_3)	46.3 µM
Magnesium sulphate $MgSO_4$)	1.5 mM	Manganese chloride ($MnCl_2$)	14.4 µM
Calcium Chloride ($CaCl_2$)	3 mM	Zinc sulphate ($ZnSO_4$)	1.4 µM
Iron (Fe) EDTA	0.1 mM	Cupric sulphate ($CuSO_4$)	0.5 µM
Ammonium sulphate $(NH_4)_2SO_4$	4 mM	Sodium Molybdate ($NaMoO_4$)	0.1 µM
Sodium Nitrate ($NaNO_3$)	8 mM		

Preparation of 5 L of modified Long Ashton nutrient solution lacking P

Stock solution	mL in 5 L	Final concentration
250 mM Potassium sulphate	40	2 mM
375 mM Magnesium sulphate	20	1.5 mM
1 M Calcium chloride	20	4 mM
110 mM Iron (Fe) EDTA	5	0.1 mM
2 M Ammonium sulphate	10	4 mM
1 M Sodium nitrate	40	8 mM
1 L Micronutrient solution	5	

Brundrett, M., Bougher, N., Dell, B., Grove, T., Malajczuk, N., 1996. Working with Mycorrhizas in Forestry and Agriculture, ACIAR monograph. Canberra.

Cavagnaro, T.R., Smith, F.A., Lorimer, M.F., Haskard, K.A., Ayling, S.M., Smith, S.E., 2001. Quantitative development of Paris-type arbuscular mycorrhizas formed between Asphodelus fistulosus and Glomus coronatum. New Phytol. 149, 105–113. https://doi.org/10.1046/j.1469-8137.2001.00001.x

McGonigle, T.P., Miller, M.H., Evans, D.G., Fairchild, G.L., Swan, J.A., 1990. A new method which gives an objective-measure of colonization of roots by vesicular arbuscular mycorrhizal fungi. New Phytol. 115, 495–501. https://doi.org/10.1111/j.1469-8137.1990.tb00476.x

Vierheilig, H., Coughlan, A.P., Wyss, U., Piché, Y., 1998. Ink and vinegar, a simple staining technique for arbuscular-mycorrhizal fungi. Appl. Environ. Microbiol. 64, 5004–5007. https://doi.org/10.1128/aem.64.12.5004-5007.1998
